# Janus kinase inhibitors vs. abatacept about safety and efficacy for patients with rheumatoid arthritis-associated interstitial lung disease: a retrospective nested case-control study

**DOI:** 10.1186/s41927-024-00374-x

**Published:** 2024-01-26

**Authors:** Atsuko Tsujii, Kentaro Isoda, Maiko Yoshimura, Akihiko Nakabayashi, Dong-Seop Kim, Tatsuya Tamada, Kurumi Yamamoto, Shiro Ohshima

**Affiliations:** 1 Department of Rheumatology, NHO Osaka Minami Medical Centre, 2-1 Kidohigashi, Kawachinagano, Osaka 586-8521 Japan; 2 Department of Clinical Research/Rheumatology, NHO Osaka Minami Medical Centre, 2-1 Kidohigashi, Kawachinagano, Osaka 586-8521 Japan; 3 Department of Clinical Research, NHO Osaka Minami Medical Centre, 2-1 Kidohigashi, Kawachinagano, Osaka 586-8521 Japan

**Keywords:** CTLA-4-Ig, Interstitial lung diseases, Janus kinase inhibitors, Retrospective study, Rheumatoid arthritis, Rheumatoid arthritis-associated interstitial lung disease

## Abstract

**Background:**

Interstitial lung disease (ILD) related to rheumatoid arthritis (RA) is among the leading causes of death and an essential prognostic factor. There is only limited evidence for the safety of anti-rheumatic drugs for patients with RA-ILD. The aim of this study is to investigate the safety and efficacy of Janus kinase inhibitors (JAKis) by comparing it with abatacept (ABT) in patients with RA-ILD.

**Methods:**

This single centre, retrospective nested case–control study enrolled patients with RA-ILD treated with JAKi or ABT. To determine the safety of the two drugs for existing ILD, we compared their drug persistency, incidence rates of pulmonary complications, and change of chest computed tomography (CT) image. For their efficacy as RA treatment, disease activity scores and prednisolone (PSL)-sparing effect were compared. We performed propensity score matching to match the groups’ patient characteristics.

**Results:**

We studied 71 patients with RA-ILD (ABT, *n* = 45; JAKi, *n* = 26). At baseline, the JAKi group had longer disease duration, longer duration of past bDMARD or JAKi use and higher usual interstitial pneumonia rate. After propensity score matching, no significant differences in patient characteristics were found between the two groups. No significant difference in the drug persistency rate for the first 2 years (ABT, 61.9%; JAKi, 42.8%; *P =* 0.256) was observed between the two matched groups. The incidence rate of pulmonary complications did not differ significantly between the two groups (*P =* 0.683). The CT score did not change after the treatment for the ABT group (Ground-glass opacities (GGO): *P* = 0.87; fibrosis: *P* = 0.78), while the GGO score significantly improved for the JAKi group (*P =* 0.03), although the number was limited (ABT: *n* = 7; JAKi: *n* = 8). The fibrosis score of the JAKi group did not change significantly.(*P* = 0.82). Regarding the efficacy for RA, a significant decrease in disease activity scores after the 1-year treatment was observed in both groups, and PSL dose was successfully tapered, although no significant differences were observed between the two drugs.

**Conclusions:**

JAKi is as safe and effective as ABT for patients with RA-ILD. JAKi can be a good treatment option for such patients.

**Supplementary Information:**

The online version contains supplementary material available at 10.1186/s41927-024-00374-x.

## Background

Rheumatoid arthritis (RA) is an autoimmune disease characterised by chronic synovitis that leads to cartilage degradation, subchondral bone erosion and eventual disability. It also involves extraarticular organs, including lungs, neurons and blood vessels [1]. Interstitial lung disease (ILD) related to RA is among the leading causes of death and an essential prognostic factor [2, 3]. RA treatment is pivotal because high disease activity of RA can be a risk factor for RA-ILD exacerbation [4]. However, therapeutic decision-making in such cases is often challenging. Some disease modifying anti-rheumatic drugs (DMARDs), including methotrexate (MTX) and leflunomide (LEF), are known for their pulmonary toxicity [5, 6]. There is also fear regarding increased infection risk in patients with existing ILD, especially those with bronchiectasis [7, 8].

Limited information is available on the safety of anti-rheumatic drugs for patients with RA-ILD. There are only a few retrospective or case studies, and treatments are often provided empirically. Presently, conventional synthetic DMARDs (csDMARDs), except MTX or LEF, and some biological DMARDs (bDMARDs), such as rituximab (RTX) or abatacept (ABT), are often used in clinical practice [9, 10].

Janus kinase inhibitors (JAKis) inhibit pathways on the proliferation of inflammatory cells and cytokine productions and are known for their immunosuppressive and anti-inflammatory effects [11]. There is solid evidence for RA, and we speculate that JAKi can also be effective for RA-ILD because lung specimens obtained from patients with RA-ILD have an increased number of immune cells [12–14]. Moreover, JAK–STAT pathways are involved in fibrosis in ILD patients [15, 16]. Therefore, we hypothesised that JAKi is a good treatment option for patients with RA-ILD. Our previous retrospective observational study reported the safety and efficacy of JAKi for patients with relatively mild RA-ILD [17]. Recently, a retrospective study in Italy showed JAKi is as effective as ABT for RA-ILD [18]. However, only a few studies have compared the safety and efficacy of JAKi with those of other anti-rheumatic drugs for patients with RA-ILD [19]. Especially, its safety for concurrent pulmonary disease remains unclear. Evidence for Japanese patients is also lacking. Therefore, in this nested case–control study, we aimed to clarify the safety of JAKi for Japanese patients with existing RA-ILD and its efficacy as a treatment for RA by comparing JAKi with ABT.

## Methods

### Patients and study design

This retrospective nested case–control study included all patients with RA-ILD who had received treatment with ABT or JAKi between April 2015 and March 2020 at our hospital. Patients receiving ABT by subcutaneous injection and intravenous drip were included. All types of JAKi were permitted in this study. All RA patients fulfilled the 1987 American College of Rheumatology (ACR) or 2010 ACR/European League against Rheumatism classification criteria [20, 21]. ILD was diagnosed by chest high-resolution computed tomography (CT) and classified as usual interstitial pneumonia (UIP), nonspecific interstitial pneumonia (NSIP) and others, according to the CT image pattern. We obtained patients’ information, including sex, age, ILD pattern, disease duration, Steinblocker stage and class, anti-cyclic citrullinated peptide antibody (ACPA), rheumatoid factor (RF), activity score 28-C-reactive protein (DAS28-CRP), modified health assessment questionnaire (mHAQ), past bDMARD or JAKi use, most recent use of bDMARDs or JAKi, MTX dose, prednisolone (PSL) dose and history of smoking and diabetes mellitus, from their previous medical records.

### Outcomes

The primary endpoint was the safety of ABT or JAKi for patients with RA-ILD, and the secondary endpoint was the efficacy for RA. The primary endpoint, safety, was evaluated according to the persistency rate of the two drugs and the incidence of pulmonary complications at 24 months after its initiation, and the change of chest CT image. Ground-glass opacities (GGO) and fibrosis were scored as previously reported for CT findings [22]. We scored the CT images at three sites, the aortic arch level, the carina, and one centimetre above the diaphragm, which represent the upper, middle, and lower lobes, respectively. The scale of the scoring is described in the supplementary Table 1. The sum of the scores was the total CT score. Pulmonary complications included serious infections requiring hospitalisation and acute exacerbation of ILD. The secondary endpoint, efficacy, was evaluated by DAS28-CRP, mHAQ and PSL dose at 12 months after the treatment initiation.

### Statistical analysis

To control for baseline patient characteristics associated with the JAKi or ABT group, propensity score matching was performed. The propensity score matching method statistically evaluates causal effects without confounding effects by mathematically transforming observational studies into randomised studies. In this study, propensity scores were calculated for each patient using a logistic regression model that included baseline covariates. The ABT and JAKi groups were matched by estimated propensity scores for each model, including age, sex, ILD pattern, disease duration and histories of bDMARDs or JAKi use. Sample matchings were performed in a 1:1 ratio without replacement based on the estimated propensity scores of each group with a caliper width set at 0.20 of the standard deviation of the propensity scores. An absolute standardised difference of < 0.10 was considered to indicate a negligible imbalance between the two groups.

Data are presented as the number of participants (percentage) or median value (interquartile range). Statistical analyses were performed using the chi-squared or Fisher’s exact test for binary data, and continuous data between the groups were compared using Wilcoxon signed-rank test or Wilcoxon test. We used multiple imputation to complete missing data. To impute the missing data, we constructed multiple regression models with potentially related variables [23]. The Kaplan–Meier method and log-rank test were performed to compare the drug persistency rate and incidences of pulmonary complications between the two groups. Statistical analyses were performed using the statistical programme JMP Pro for Windows (version 13.0, SAS Institute Inc., Cary, NC, USA). A *P* value of < 0.05 was considered significant.

## Results

### Patients’ baseline characteristics

We included 71 patients (ABT, *n* = 45; JAKi, *n* = 26). Table 1 shows the baseline characteristics of the patients included. Regarding the ILD pattern, UIP pattern rate was significantly higher in the ABT group than in the JAKi group [ABT, *n* = 23 (51.1%); JAKi, *n* = 4 (15.4%); *P =* 0.002]. The disease duration at treatment initiation was significantly longer in the JAKi group than in the ABT group [ABT, 6 (3–18) years; JAKi, 17 (7–25) years; *P =* 0.024]. The rate of past bDMARDs or JAKi use was significantly higher in the JAKi group than in the ABT group [ABT, *n* = 13 (28.9%); JAKi, *n* = 21 (80%); *P <* 0.001].

**Table 1:** Comparison of patient’s clinical characteristics at baseline between the ABT and JAKi groups

	ABT	JAKi	***p*** value
Number of patients	45	26	
Gender, female (%)	29 (64.4)	18 (69.2)	0.797
Age, years	74 (71–81)	74 (70–79)	0.384
ILD, n (%)			0.002
UIP	23 (51.1)	4 (15.4)	
NSIP	22 (48.9)	20 (76.9)	
Unclassifiable	0 (0.0)	2 (7.7)	
ABT, n (%)			
Subcutaneous injection	36 (0.8)	-	
Intravenous drip	9 (0.2)	-	
JAKi, n (%)			
Tofacitinib	-	16 (61.5)	
Baricitinib	-	10 (38.5)	
Disease duration, years	6 (3–18)	17 (7–25)	0.024
Stage ≥ III, n (%)	20 (44.4)	14 (53.9)	0.471
Class ≥ 3, n (%)	4 (8.9)	1 (3.9)	0.646
ACPA positive, n (%)	40 (88.9)	26(100)	0.151
ACPA ≥ 100, n (%)	28 (62.2)	19 (73.1)	0.439
RF positive, n (%)	40 (88.9)	26 (100)	0.151
RF ≥ 100, n (%)	28 (62.2)	19 (73.1)	0.439
DAS28-CRP	4.37 (3.91–5.21)	4.35 (3.26–5.58)	0.919
mHAQ	0.81 (0.13–1.13)	0.63 (0.10–1.41)	0.852
Past use of bDMARDs or JAKi, n (%)	13 (28.9)	21 (80.8)	< 0.001
Most recent bDMARDs or JAKi, n (%)			0.057
IL6i	6 (46.2)	8 (33.3)	
CTLA4-ig	-	7 (38.1)	
TNFi	6 (46.2)	6 (28.6)	
JAKi	1 (7.7)	0 (0.0)	
Methotrexate, n (%)	14 (31.1)	6 (23.1)	0.588
Prednisolone, n (%)	31 (68.9)	19 (73.1)	0.792
Prednisolone dose, mg/day	4.0 (0.0–5.0)	4.5 (2.5–6.0)	0.405
Smoking, n (%)			0.381
Never	31 (68.9)	18 (69.2)	
Ex-smoker	8 (17.8)	2 (7.7)	
Current smoker	6 (13.3)	6 (23.1)	
Diabetes mellitus, n (%)	7 (15.6)	3 (11.5)	0.736

Data are presented as number of patients (%) or the median value (interquartile range)

ABT: abatacept; JAKi: Janus kinase inhibitor; ILD: interstitial lung disease; UIP: usual interstitial pneumonia; NSIP: nonspecific interstitial pneumonia; ACPA: anti-cyclic citrullinated peptide antibody; RF: rheumatoid factor; DAS28-CRP: disease activity score 28-C-reactive protein; mHAQ: modified health assessment questionnaire; bDMARD: biological disease modifying anti-rheumatic drugs; IL6i: anti-inteleukin-6 inhibitors; CTLA4-ig: cytotoxic T lymphocyte-associated antigen-4-immunoglobulin; TNFi: anti-TNFα inhibitors

**Fig. 1:**
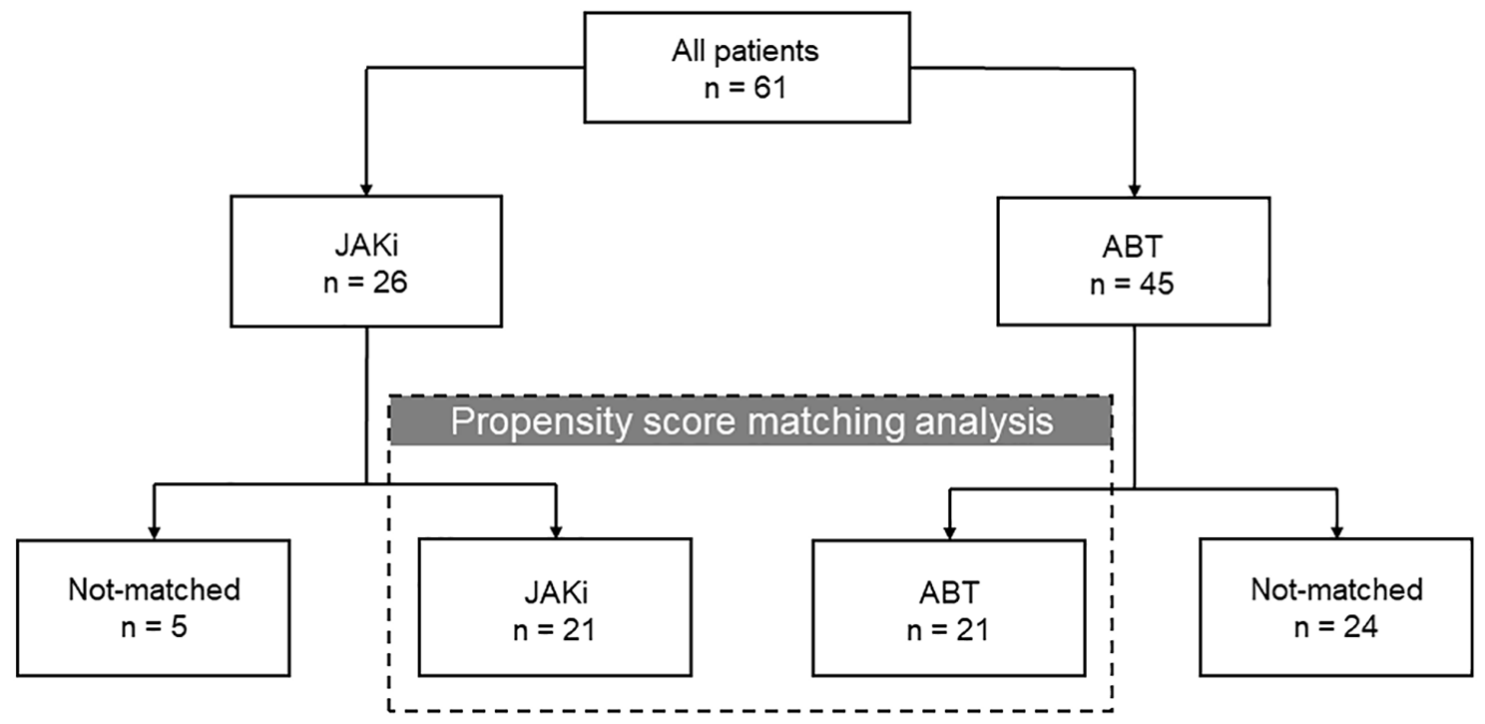
Flow diagram of the study Sixty-one patients (JAKi: 26 patients, ABT: 45 patients) were recruited. After a propensity score matching analysis, 21 patients were assigned to each group JAKi: Janus Kinase inhibitor; ABT: abatacept

Among 71 patients enrolled, 21 were selected for the JAKi and ABT group after propensity score matching (Fig. 1). Table 2 shows the characteristics of the patients selected by propensity score matching analysis. The clinical characteristics, including the ILD pattern, disease duration and rate of past bDMARD or JAKi use, were not significantly different between the two matched groups. The percentage of the NSIP pattern was higher in our study than in previous studies [ABT, *n* = 18 (85.7%); JAKi, *n* = 17 (80.9%)] [24, 25].

**Table 2:** Comparison of patients’ baseline clinical characteristics by propensity score matching analysis

	ABT	JAKi	***p*** value
Number of patients	21	21	
Gender, female (%)	14 (66.7)	14 (66.7)	N.S.
Age, years	72 (74–80)	74 (71–79)	N.S.
ILD, n (%)			N.S.
UIP	3 (14.3)	4 (19.1)	
NSIP	18 (85.7)	17 (80.9)	
ABT, n (%)			
Subcutaneous injection	3 (14.3)	-	
Intravenous drip	18 (86.7)	-	
JAKi, n (%)			
Tofacitinib	-	14 (66.7)	
Baricitinib	-	7 (33.3)	
Disease duration, years	6 (4–19)	16 (7–26)	N.S.
Stage ≥ III, n (%)	11 (52.3)	12 (57.1)	N.S.
Class ≥ 3, n (%)	3 (14.3)	1 (4.8)	N.S.
ACPA positive, n (%)	18 (85.1)	21 (100)	N.S.
ACPA ≥ 100, n (%)	11 (52,3)	15 (71.4)	N.S.
RF positive, n (%)	18 (85.7)	21 (100)	N.S.
RF ≥ 100, n (%)	14 (66.7)	14 (66.7)	N.S.
DAS28-CRP	4.37 (3.04–5.02)	4.58 (3.50–5.80)	N.S.
mHAQ	0.75 (0.00–1.07)	0.38 (0.07–1.50)	N.S.
Past use of bDMARDs or JAKi, n (%)	9 (42.9)	16 (76.2)	N.S.
Most recent bDMARDs or JAKi, n (%)			N.S.
IL6i	4 (44.4)	7 (43.8)	
CTLA4-ig	-	5 (31.3)	
TNFi	5 (55.6)	4 (24.0)	
JAKi	0 (0.0)	0 (0.0)	
Methotrexate, n (%)	10 (47.6)	5 (23.8)	N.S.
Prednisolone, n (%)	17 (81.0)	14 (66.7)	N.S.
Prednisolone dose, mg/day	5.0 (2.3–5.0)	4.8 (0.0–6.0)	N.S.
Smoking, n (%)			N.S.
Never	17 (81.0)	14 (66.7)	
Ex-smoker	2 (9.5)	5 (23.8)	
Current smoker	2 (9.5)	2 (9.5)	
Diabetes mellitus, n (%)	4 (19.1)	2 (9.5)	N.S.

Data are presented as number of patients (%) or the median value (interquartile range)

ABT: abatacept; JAKi: Janus kinase inhibitors; ILD: interstitial lung disease; UIP: usual interstitial pneumonia; NSIP: nonspecific interstitial pneumonia; ACPA: anti-cyclic citrullinated peptide antibody; RF: rheumatoid factor; DAS28-CRP: disease activity score 28-C-reactive protein; mHAQ: modified health assessment questionnaire; bDMARD: biological disease modifying anti-rheumatic drugs; IL6i: anti-interleukin-6 inhibitors; CTLA4-ig: cytotoxic T lymphocyte-associated antigen-4-immunoglobulin; TNFi: anti-TNFα inhibitors. N.S.: not significant

### The severity of ILD

To evaluate the severity of ILD in matched patients, we assessed KL-6 and CT images. KL-6 at the beginning of the treatment was evaluated. The median value of KL-6 was 323.5 U/mL for ABT, and 395 U/mL for JAKi. KL-6 was not significant between the two groups (*P =* 0.43). As for chest CT image, we evaluated CT scores as described in the method. The median GGO score for ABT and JAKi was 3.5 and 4.0, respectively (*n* = 19; *n* = 17; *P =* 0.004). The median fibrosis score for ABT and JAKi was 2.5 and 4.0, respectively (*P =* 0.20). The details of CT scores are summarized in supplementary Table 2.

### The drug persistency rate

**Fig. 2:**
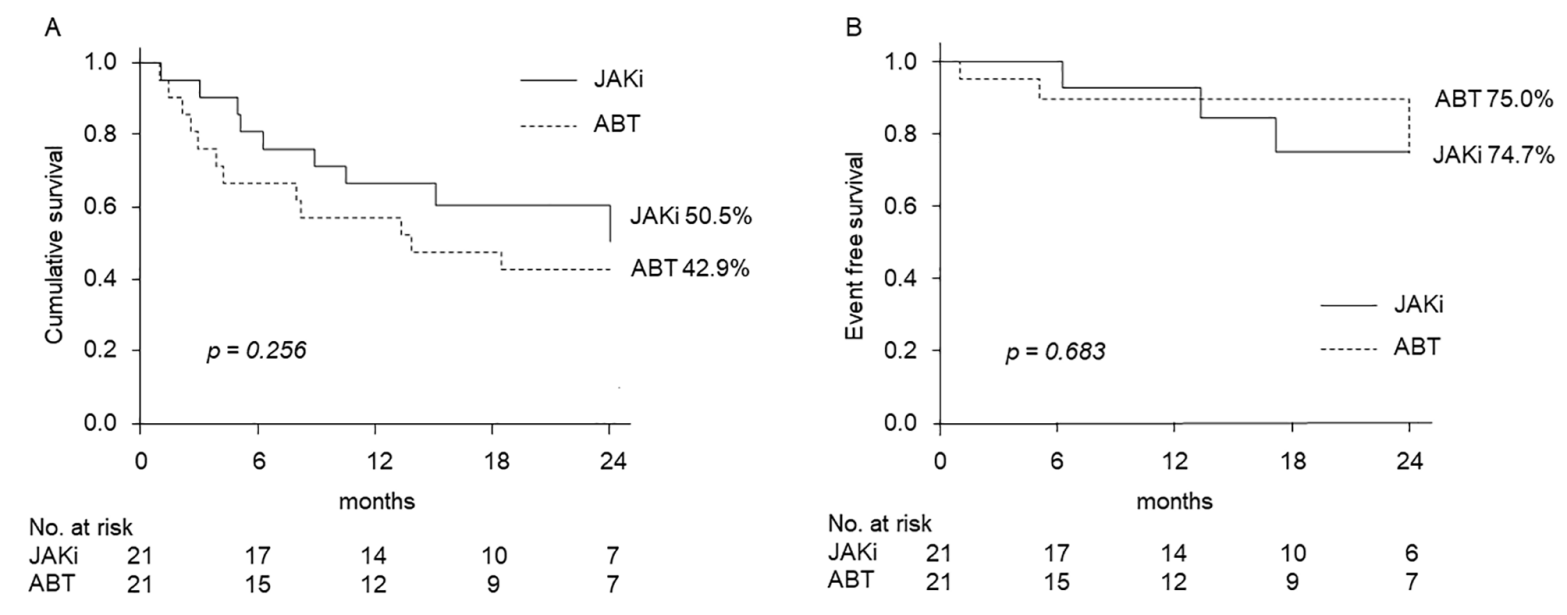
The Kaplan–Meier curves of the persistency rates and event-free survival rates of the two drugs **(A)** The Kaplan–Meier curves of the persistency rate of the two drugs. **(B)** The Kaplan–Meier curves of the event-free survival of the two drugs The persistency rates at 2 years after treatment initiation were 42.9% and 50.5% for ABT and JAKi, respectively. The event-free survival rates at 2 years after treatment initiation were 75.0% and 74.7%, respectively. There was no significant difference in the rates between the two groups (A, *P =* 0.256; B, *P =* 0.683) JAKi: Janus kinase inhibitor; ABT: abatacept

Kaplan–Meier curves were plotted to compare the persistency rate between the two drugs (Fig. 2-A). The persistency rate at 24 months after the initiation was not significantly different between the two drugs [ABT, 42.9%; JAKi, 50.5%; *P =* 0.256]. Thirteen patients (61.9%) in the ABT group and nine patients (42.8%) in the JAKi group discontinued the drug by the end of the follow-up period. The reasons for discontinuation included inefficacy and adverse effects (Table 3). Inefficacy means lack of improvement of DAS28-CRP. The ABT group included nine cases of inefficacy and four cases of adverse effects, whereas the JAKi group included four cases of inefficacy and five cases of adverse effects. The reason for the discontinuation did not differ between the two groups (*P =* 0.284). The adverse effects in the ABT group included skin rash, drop in blood pressure, dizziness and chronic obstructive pulmonary disease. The adverse effects in the JAKi group included infections (one case of herpes zosters and one case of herpes labialis), haematological problem, organising pneumonia and cancer (gastric cancer). There were no deaths during the study period.

**Table 3:** The rate of drug discontinuation and its reasons

	ABT (*n* = 21)	JAKi (*n* = 21)
Lack of effect, n (%)	9 (42.9)	4 (19.0)
Adverse events, n (%)	4 (19.0)	5 (23.8)
Infections, n (%)	0 (0.0)	2 (9.5)
Lung involvement, n (%)	0 (0.0)	1 (4.8)
Leukopenia, n (%)	0 (0.0)	1 (4.8)
Neoplasia n (%)	0 (0.0)	1 (4.8)
Others, n (%)	4 (19.0)	0 (0.0)

Data are presented as number of patients (%)

ABT: abatacept; JAKi: Janus kinase inhibitors; Infections: herpes zosters and herpes labialis; Others: skin rash, drop in the blood pressure, dizziness, chronic obstructive pulmonary disease

### Change in chest CT image

**Fig. 3:**
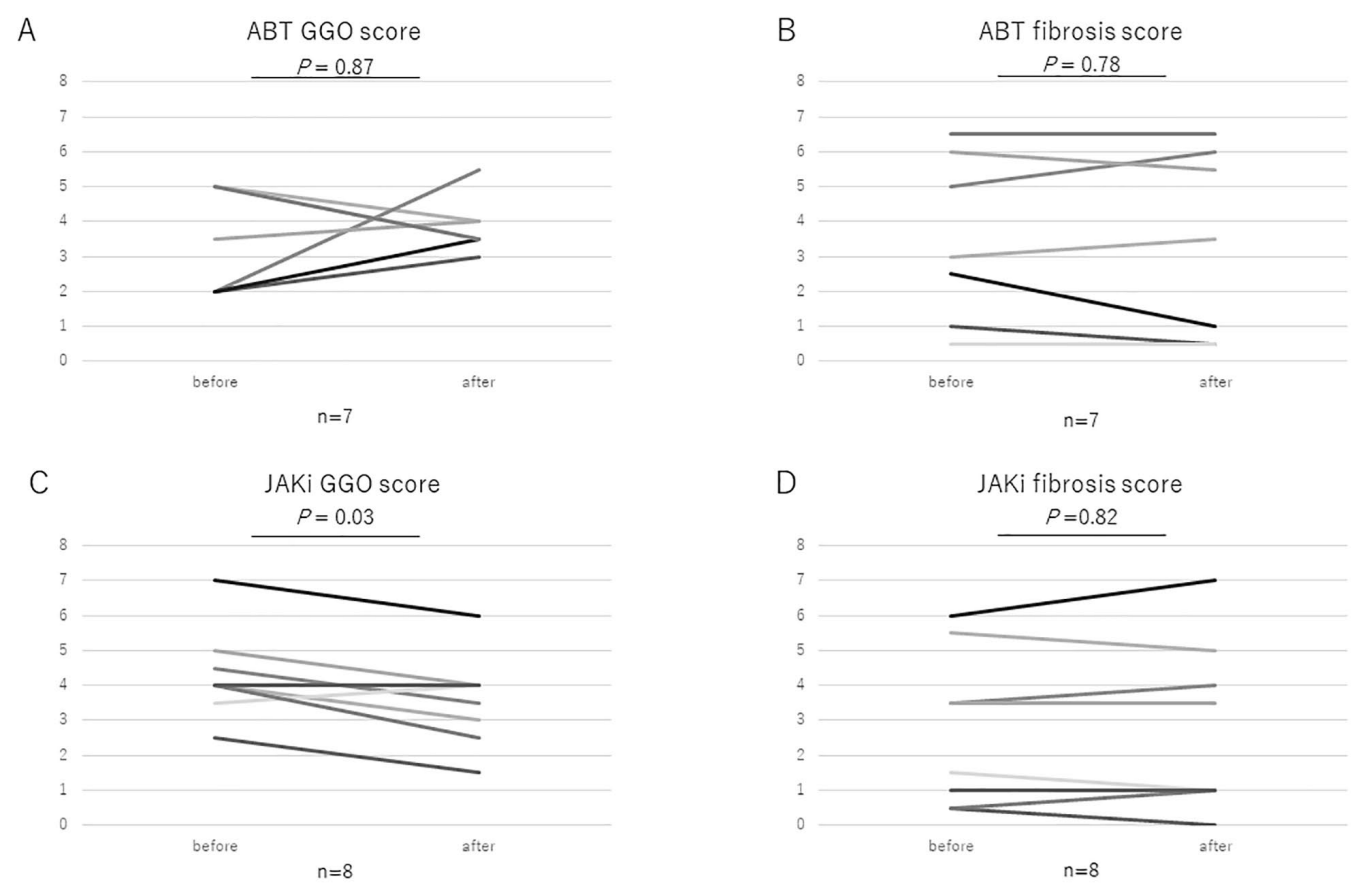
The change of the CT score after treatment initiation **(A)** GGO score of the ABT group (*n* = 7). **(B)** Fibrosis score of the ABT group (*n* = 7). **(C)** GGO score of the JAKi group (*n* = 8). **(D)** Fibrosis score of the JAKi group (*n* = 8) Change in the CT score. CT score was evaluated after treatment initiation. The median of the period between treatment initiation and the follow-up CT was 13 and 14.5 months for ABT and JAKi, respectively GGO; grass ground opacity; CT: computer tomography; JAKi: Janus kinase inhibitor; ABT: abatacept

The change in CT score after the treatment initiation was evaluated. Some of the CT images after the treatment initiation were lacking among the patients. Except for such cases and cases that stopped treatment, seven and eight cases were evaluated for the ABT and JAKi groups respectively. The median of the period between the start of the treatment and the follow-up CT was 13 and 14.5 months for the ABT and JAKi groups respectively. Figure 3 shows the result of the CT score. The CT score did not change after the treatment in the ABT group (Ground-glass opacities (GGO): *P* = 0.87; fibrosis: *P* = 0.78), while the GGO score significantly improved after starting the treatment for the JAKi group (GGO: *P* = 0.03). The fibrosis score of the JAKi group did not change significantly.(*P* = 0.82).

### Pulmonary complications

Figure 2-B shows the Kaplan–Meier curves for the incidence of pulmonary complications. The incidence rate did not differ significantly between the two groups [ABT, 25.0%; JAKi, 25.3%; *P =* 0.683]. Pulmonary complications occurred in three and two patients who used ABT and JAKi, respectively (Table 4). The pulmonary complications included two cases of pneumonia and one case of empyema for the ABT group, and one case of influenza pneumonia and one case of organising pneumonia for the JAKi group. All complications were cured after hospitalisation. There was no case of acute exacerbation of the ILD in both groups.

**Table 4:** Lung involvement

	Occurrence	Lung involvement
ABT (*n* = 21)	3 (14.3%)	Pneumonia (*n* = 2) Empyema (*n* = 1)
JAKi (*n* = 21)	2 (9.5%)	Influenza pneumonia (*n* = 1) Organized pneumonia (*n* = 1)

ABT: abatacept; JAKi: Janus kinase inhibitors

### Efficacy for RA

**Fig. 4:**
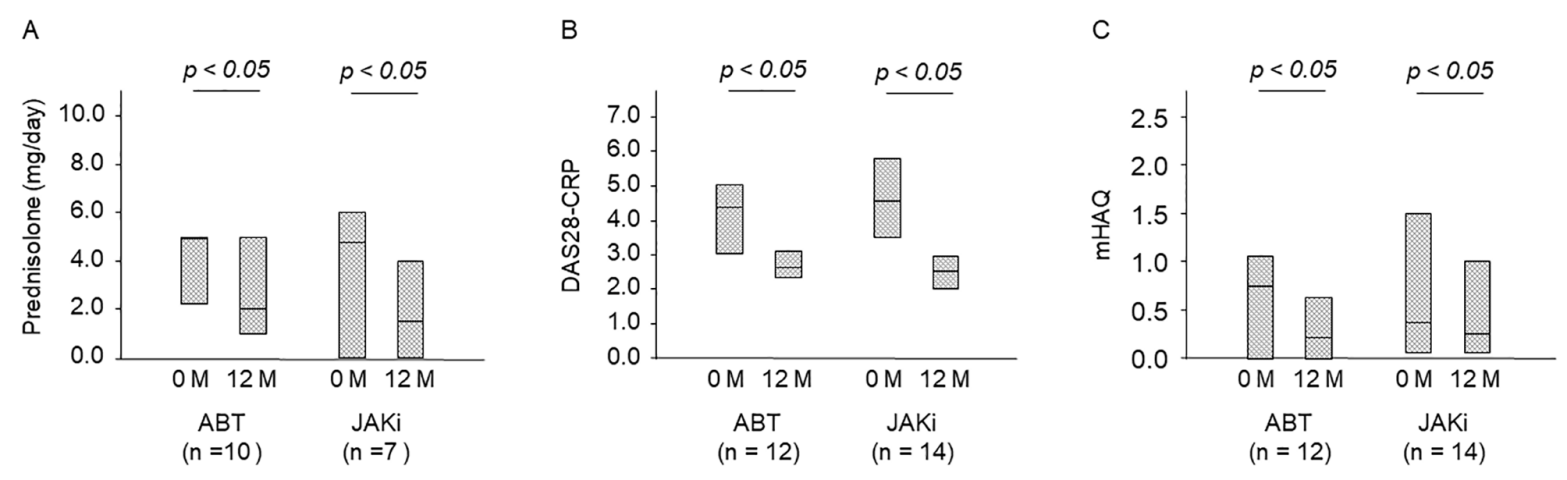
Clinical evaluation at 12 months after treatment initiation of the two drugs Changes in **(A)** prednisolone dosage, **(B)** DAS28-CRP and **(C)** mHAQ responses from the drug initiation to 12 months after. In both drug groups, there was a significant change in prednisolone dosage, DAS28-CRP and mHAQ after 12 months of initiation, but no significant difference was observed between the two drugs (**A**, *P =* 0.057; **B**, *P =* 0.551; **C**, *P =* 0.488) JAKi: Janus kinase inhibitor; ABT: abatacept; DAS28-CRP: disease activity score 28-C-reactive protein; mHAQ: modified health assessment questionnaire

Figure 4 shows the change in DAS28-CRP, mHAQ and PSL dose after 12 months of treatment. DAS28-CRP significantly reduced after 12 months of treatment [ABT, 4.37 (3.04–5.02) to 2.63 (2.35–3.11), *P* < 0.05; JAKi, 4.58 (3.50–5.80) to 2.55 (2.02–2.94), *P* < 0.05]. mHAQ also significantly improved after 12 months of treatment [ABT, 0.75 (0–1.07) to 0.21 (0–0.64), *P* < 0.05; JAKi, 0.38 (0.07–1.5) to 0.21 (0.07–0.64), *P* < 0.05]]. Moreover, the PSL dose was significantly reduced in both groups [ABT, 5.0 (2.3–5.0) mg/day to 2.0 (1.0–5.0) mg/day, *P* < 0.05; JAKi, 4.8 (0.0–6.0) mg/day to 1.50 (0.0–4.0) mg/day, *P* < 0.05]. Between the two drug groups, there was no difference in DAS28-CRP improvement, mHAQ improvement and PSL reduction (*P =* 0.057, *P =* 0.551 and *P =* 0.488, respectively).

## Discussion

This retrospective nested case–control study compared JAKi with ABT to clarify the safety and efficacy of JAKi in Japanese patients with RA-ILD. The results showed that there were no differences in the drug persistency rate and pulmonary complication rates between JAKi and ABT. The reason for drug discontinuation did not differ significantly between the two groups, and no exacerbation of the existing ILD was reported in either group. The GGO scores in terms of the change of the chest CT significantly improved for the JAKi group while they remained for the ABT group. The fibrosis scores did not change in both groups. JAKi was as effective as ABT for treating arthritis, which enabled us to taper the PSL dosage. To the best of our knowledge, this is the first study to show that JAKi can be a safe and effective option for patients with RA-ILD by comparing it with ABT.

RA treatments have progressed considerably; in fact, remission or low disease activity of RA can be achieved in many patients using a variety of drugs, but no standard treatments have been established for RA-ILD yet. It is one of the leading causes of death and an essential prognostic factor [2, 3]. Choosing an anti-rheumatic drug is often challenging for several reasons. First, some anti-rheumatic drugs can cause drug-induced pneumonia or exacerbate existing ILD. MTX and LEF are known to induce drug-induced pneumonia [5, 6]. As for bDMARDs, ILD exacerbation is reportedly caused by TNFi [26, 27] and anti-IL-6 drugs [28, 29]. Additionally, there is also fear for an increased risk of infections due to bDMARDs [7, 8].

Several suggestions were presented about the treatment of patients with RA-ILD [9, 30], but there is no established evidence for the safety of anti-rheumatic drugs for patients with RA-ILD; thus, treatments are often given empirically. As for the patients selected for this study, ABT or JAKi was chosen according to each physician’s choice, considering each patient’s conditions, including DAS28, ILD, and other safety concerns. There is growing evidence about the safety of ABT and RTX for patients with RA-ILD. The safety of abatacept for patients with RA-ILD has been shown in some case reports and retrospective studies [31–33]. A multicentre, retrospective observational study in Spain showed the efficacy of ABT for arthritis without exacerbating the existing ILD in 263 patients with RA-ILD [31]. One possible mechanism is that ABT can prevent exacerbation of ILD by blocking T cells, as shown in animal models [32]. Additionally, infectious risk with ABT is lower than the other bDMARDs, which is also good for patients with RA-ILD. Likewise, the safety of RTX is also reported by some studies [34, 35]. Currently in Japan, ABT is commonly used because RTX is not allowed for RA patients. Presently, there is a lack of solid evidence; thus, the question ‘which anti-rheumatic drug can be used safely for patients with RA-ILD’ remains unanswered.

JAKi inhibits the JAK–STAT pathways and broadly blocks the pathways on inflammation [11]. In the lung specimen from patients with RA-ILD, numerous CD4 positive T cells are present; thus, it can be speculated that JAKi can suppress the activation of alveolar inflammatory cells by inhibiting multiple cytokines via the JAK–STAT pathway [14, 36]. Moreover, there is growing evidence that the JAK–STAT pathway is activated under a number of profibrotic cytokines and engages in fibrotic processes. Especially, the JAK2–STAT3 pathways are predominant in ILD, and phosphorylation of the pathway leads to cellular fibrotic processes, including epithelial/fibroblast to mesenchymal transition [15]. Another study has shown that JAK2–STAT5A & B pathway is involved in fibrosis in RA-ILD [16]. A basic research study showed that tofacitinib successfully suppressed the progression of ILD in SKG mice, facilitated the expansion of myeloid-derived suppressor cells in the lung and ameliorated ILD by suppressing the proliferation of T cells and differentiation of Th17 cells [37]. From these findings, we speculate that JAKi can inhibit the progression of RA-ILD by blocking these processes.

In the clinical field, some case reports suggest the efficacy of JAKi in ILD. In MDA5 antibody-positive dermatomyositis, several case reports show the efficacy of tofacitinib [38–40]. As for RA-ILD, only a few case reports or retrospective studies showed the efficacy of JAKi. JAKi as treatment for arthritis without exacerbating the existing ILD was effective in four patients [41]. Moreover, our previous observational study showed efficacy for arthritis without causing exacerbation of ILD or increasing the risk for infection in relatively mild patients with RA-ILD [17]. Comparing JAKi with RTX, no significant difference in hospitalisation and mortality rates was reported [19]. Additionally, a retrospective study in Italy recently showed that treatment with JAKi or ABT was related to stability or improvement of RA-ILD in many of the patients [18]. As mentioned, there is growing evidence of JAKi use for patients with RA-ILD, but there is no solid evidence yet, especially in terms of its safety. The present study showed no difference in the drug persistency and pulmonary complication rates between ABT and JAKi. We even demonstrated that the GGO scores improved significantly for the JAKi group, while the scores did not change for the ABT group, although the number evaluated was quite small for the change of the chest CT image. Our result provides information about the safety of JAKi in patients with RA-ILD and indicates that JAKi can be a safe treatment option in these patients, which can be helpful in clinical practice.

Our research also showed the efficacy of JAKi for arthritis without exacerbating ILD. There is quite solid evidence showing the efficacy of JAKi for RA without extraarticular lesions [12, 13]. However, its effectiveness for cases of arthritis with pulmonary complications has rarely been reported. The treatment of the arthritis is essential because the high disease activity of RA can be a risk factor for ILD exacerbation [4]. Therefore, our result provides clinically important evidence. Especially, JAKi can be a good treatment option for patients who cannot use ABT for some reasons. For example, ABT is reported to be less effective in ACPA-negative patients [42, 43]; thus, JAKi can be a preferable option for such patients. JAKi. can be also used for patients for whom ABT was not effective or ABT cannot be used because of adverse events.

Another clinically important result is that JAKi helped in tapering the PSL dose in patients with RA-ILD. In addition to the numerous side effects of PSL, such as diabetes or osteoporosis, PSL usage increases the risk of developing progressive fibrosing interstitial lung disease which is associated with increased mortality in patients with ILD [44]. Additionally, PSL can increase the risk of infections, which are also a risk factor for ILD exacerbation [45]. For these reasons, tapering of the PSL dose not only can improve the patients with RA-ILD quality of life by getting rid of the troublesome side effects, but also can improve their prognosis.

In this study, the percentage of NSIP patterns was much higher (> 80%) than those previously reported [24, 25]. One plausible reason for this is that physicians avoided using JAKi or ABT for UIP patterns because of higher risks of exacerbation or infections in UIP patterns. Additionally, as for the severity of ILD, the median value of KL-6 was less than 400 U/mL. The cut-off value of KL-6 is less than 500 U/mL. Therefore in these respects, this study included less severe patients. We should monitor patients for ILD exacerbation because ILD exacerbation possibly caused by JAKi has been reported [46]. In addition, we should pay close attention to cases of pulmonary infections due to the increased risk of infectious disease by JAKi [12], especially in patients with bronchiectasis or a UIP pattern.

Our study has several strengths; To best of our knowledge, this study is the first to show safety of JAKi for patients with RA-ILD by comparing with ABT. Additionally, this study included 26 and 45 Japanese patients with RA-ILD for the JAKi and ABT groups, respectively, which is greater than the number of cases analysed previous studies. Moreover, by selecting patients with propensity score matching, there is little influence of confounding factors.

This study has several limitations. First, this retrospective study was conducted at a single centre, and the number of the patients is limited. Second, some of the follow-up CTs were lacking, so the number of follow-up CTs is even more limited. Third, we did not evaluate the lung function by respiratory function test or 6 min walk distance to investigate the efficacy of the treatments for ILD because of a lack of data. Finally, because of the limited number of patients included, we did not conduct a subgroup analysis; thus, the question ‘who among the patients will benefit more from the JAKi treatment’, or ‘which JAKi type is the most effective’ has remained unanswered.

We speculate that JAKi is effective especially for those with NSIP patterns whose main pathophysiology is the invasion of inflammatory cells. As for differences among the JAKi types, it is difficult to determine which JAKi is the best for patients with RA-ILD. Regarding efficacy, pan-JAK inhibitors, rather than JAK1 selective inhibitors, may work more effectively, especially for cases with a UIP pattern, considering a predominant role of JAK2 in fibrosis of ILDs. However, in terms of the risks for infection, the use of JAK1 selective inhibitors may be safer. To clarify these questions, further studies, especially multicentre, prospective cohort studies, are necessary in the future.

## Conclusions

JAKi is a relatively safe treatment option for Japanese patients with RA-ILD, after comparing it with ABT. Our study data could be useful as we provide treatment options for patients with RA-ILD, which can affect the patients’ prognosis. Our results suggest that JAKi can improve the prognosis of patients with RA-ILD.

### Electronic supplementary material

Below is the link to the electronic supplementary material.

Supplementary Material 1

## Data Availability

All data generated or analysed during this study are included in this published article.

## Abbreviations

ABT: abatacept
JAKi: Janus kinase inhibitor
ILD: interstitial lung disease
UIP: usual interstitial pneumonia
NSIP: nonspecific interstitial pneumonia
ACPA: anti-cyclic citrullinated peptide antibody
RA: rheumatoid arthritis
RF: rheumatoid factor
DAS28-CRP: disease activity score 28-C-reactive protein
mHAQ: modified health assessment questionnaire
bDMARD: biological disease modifying anti-rheumatic drugs
GGO: ground-glass opacities
IL6i: anti-inteleukin-6 inhibitors
CTLA4-ig: cytotoxic T lymphocyte-associated antigen-4-immunogloblin
TNFi: anti-TNFα inhibitors

## References

[1] Rajeshwari B, Kumar S (2023). Rheumatoid neuropathy: a brief overview. Cureus.

[2] Olson AL, Swigris JJ, Sprunger DB, Fischer A, Fernandez-Perez ER, Solomon J (2011). Rheumatoid arthritis-interstitial lung disease-associated mortality. Am J Respir Crit Care Med.

[3] Kakutani T, Hashimoto A, Tominaga A, Kodama K, Nogi S, Tsuno H (2020). Related factors, increased mortality and causes of death in patients with rheumatoid arthritis-associated interstitial lung disease. Mod Rheumatol.

[4] Akiyama M, Kaneko Y, Yamaoka K, Kondo H, Takeuchi T (2016). Association of disease activity with acute exacerbation of interstitial lung disease during tocilizumab treatment in patients with rheumatoid arthritis: a retrospective, case-control study. Rheumatol Int.

[5] Saravanan V, Kelly CA (2004). Reducing the risk of methotrexate pneumonitis in rheumatoid arthritis. Rheumatology.

[6] Conway R, Low C, Coughlan RJ, O’Donnell MJ, Carey JJ (2016). Leflunomide use and risk of lung disease in rheumatoid arthritis: a systematic literature review and metaanalysis of randomized controlled trials. J Rheumatol.

[7] Wolfe F, Caplan L, Michaud K (2006). Treatment for rheumatoid arthritis and the risk of hospitalization for pneumonia: associations with prednisone, disease-modifying antirheumatic drugs, and anti-tumor necrosis factor therapy. Arthritis Rheum.

[8] Sakai R, Komano Y, Tanaka M, Nanki T, Koike R, Nagasawa H (2012). Time-dependent increased risk for serious infection from continuous use of tumor necrosis factor antagonists over three years in patients with rheumatoid arthritis. Arthritis Care Res (Hoboken).

[9] Holroyd CR, Seth R, Bukhari M, Malaviya A, Holmes C, Curtis E (2019). The British Society for Rheumatology Biologic DMARD safety guidelines in inflammatory arthritis-executive summary. Rheumatology(Oxford).

[10] Solomon JJ, Swigris JJ, Kreuter M, Polke M, Aronson K, Hoffmann-Vold AM (2022). The attitudes and practices of physicians caring for patients with rheumatoid arthritis-associated interstitial lung disease: an international survey. Rheumatology.

[11] O’Shea JJ, Schwartz DM, Villarino AV, Gadina M, McInnes IB, Laurence A (2015). The JAK-STAT pathway: impact on human disease and therapeutic intervention. Annu Rev Med.

[12] Taylor PC, Keystone EC, van der Heijde D, Weinblatt ME, Del Carmen Morales L, Reyes Gonzaga J (2017). Baricitinib versus placebo or adalimumab in rheumatoid arthritis. N Engl J Med.

[13] Fleischmann R, Kremer J, Cush J, Schulze-Koops H, Connell CA, Bradley JD (2012). Placebo-controlled trial of tofacitinib monotherapy in rheumatoid arthritis. N Engl J Med.

[14] Turesson C, Matteson EL, Colby TV, Vuk-Pavlovic Z, Vassallo R, Weyand CM (2005). Increased CD4 + T cell infiltrates in rheumatoid arthritis-associated interstitial pneumonitis compared with idiopathic interstitial pneumonitis. Arthritis Rheum.

[15] Xin P, Xu X, Deng C, Liu S, Wang Y, Zhou X (2020). The role of JAK/STAT signaling pathway and its inhibitors in diseases. Int Immunopharmacol.

[16] Wang S, Liu M, Li X, Zhang J, Wang F, Zhang C (2022). Canonical and noncanonical regulatory roles for JAK2 in the pathogenesis of rheumatoid arthritis-associated interstitial lung disease and idiopathic pulmonary fibrosis. FASEB J.

[17] Isoda K, Tsujii A, Harada Y, Yoshimura M, Matsuoka H, Nakabayashi A (2022). Efficacy and safety of Janus kinase inhibitors for rheumatoid arthritis associated interstitial lung disease. Clin Rheumatol Rel Res.

[18] Tardella M, Di Carlo M, Carotti M, Ceccarelli L, Giovagnoni A, Salaffi F (2022). A retrospective study of the efficacy of JAK inhibitors or abatacept on rheumatoid arthritis-interstitial lung disease. Inflammopharmacology.

[19] Cronin O, McKnight O, Keir L, Ralston SH, Hirani N, Harris H (2021). A retrospective comparison of respiratory events with JAK inhibitors or rituximab for rheumatoid arthritis in patients with pulmonary disease. Rheumatol Int.

[20] Aletaha D, Neogi T, Silman AJ, Funovits J, Felson DT, Bingham CO (2010). 2010 rheumatoid arthritis classification criteria: an American College of Rheumatology/European League against Rheumatism collaborative initiative. Arthritis Rheum.

[21] Arnett FC, Edworthy SM, Bloch DA, McShane DJ, Fries JF, Cooper NS (1988). The American Rheumatism Association 1987 revised criteria for the classification of rheumatoid arthritis. Arthritis Rheum.

[22] Kazerooni EA, Martinez FJ, Flint A, Jamadar DA, Gross BH, Spizarny DL (1997). Thin-section CT obtained at 10-mm increments versus limited three-level thin-section CT for idiopathic pulmonary fibrosis: correlation with pathologic scoring. AJR Am J Roentgenol.

[23] Schäfer J, Strimmer K (2005). A shrinkage approach to large-scale covariance matrix estimation and implications for functional genomics. Stat Appl Genet Mol Biol.

[24] Kim EJ, Elicker BM, Maldonado F, Webb WR, Ryu JH, Van Uden JH (2010). Usual interstitial pneumonia in rheumatoid arthritis-associated interstitial lung disease. Eur Respir J.

[25] Lee HK, Kim DS, Yoo B, Seo JB, Rho JY, Colby TV (2005). Histopathologic pattern and clinical features of rheumatoid arthritis-associated interstitial lung disease. Chest.

[26] Nakashita T, Ando K, Kaneko N, Takahashi K, Motojima S (2014). Potential risk of TNF inhibitors on the progression of interstitial lung disease in patients with rheumatoid arthritis. BMJ Open.

[27] Kang EH, Jin Y, Desai RJ, Liu J, Sparks JA, Kim SC (2020). Risk of exacerbation of pulmonary comorbidities in patients with rheumatoid arthritis after initiation of abatacept versus TNF inhibitors: a cohort study. Semin Arthritis Rheum.

[28] Manfredi A, Sebastiani M, Cassone G, Colaci M, Sandri G, Ferri C (2018). Tocilizumab for the treatment of patients with rheumatoid arthritis and interstitial lung diseases: a case series. Clin Exp Rheumatol.

[29] Kawashiri SY, Kawakami A, Sakamoto N, Ishimatsu Y, Eguchi K (2012). A fatal case of acute exacerbation of interstitial lung disease in a patient with rheumatoid arthritis during treatment with tocilizumab. Rheumatol Int.

[30] 30, Conway R, Corcoran L, Nikiphorou E. Efficacy and safety of conventional synthetic, biologic, and targeted synthetic disease-modifying antirheumatic drugs in rheumatoid arthritis-interstitial lung disease: a narrative review. Indian J Rheumatol. 2021;16(Suppl 1):92–100.

[31] Fernández-Díaz C, Castañeda S, Melero-González RB, Ortiz-Sanjuán F, Juan-Mas A, Carrasco-Cubero C (2020). Abatacept in interstitial lung disease associated with rheumatoid arthritis: national multicenter study of 263 patients. Rheumatology.

[32] Tardella M, Di Carlo M, Carotti M, Giovagnoni A, Salaffi F (2021). Abatacept in rheumatoid arthritis-associated interstitial lung disease: short-term outcomes and predictors of progression. Clin Rheumatol.

[33] Nakashita T, Ando K, Takahashi K, Motojima S (2016). Possible effect of abatacept on the progression of interstitial lung disease in rheumatoid arthritis patients. Respir Investig.

[34] Md Yusof MY, Kabia A, Darby M, Lettieri G, Beirne P, Vital EM (2017). Effect of rituximab on the progression of rheumatoid arthritis-related interstitial lung disease: 10 years’ experience at a single centre. Rheumatology.

[35] Vadillo C, Nieto MA, Romero-Bueno F, Leon L, Sanchez-Pernaute O, Rodriguez-Nieto MJ (2020). Efficacy of Rituximab in slowing down progression of rheumatoid arthritis-related interstitial lung disease: data from the NEREA registry. Rheumatology.

[36] Winthrop KL (2017). The emerging safety profile of JAK inhibitors in rheumatic disease. Nat Rev Rheumatol.

[37] Sendo S, Saegusa J, Yamada H, Nishimura K, Morinobu A (2019). Tofacitinib facilitates the expansion of myeloid-derived suppressor cells and ameliorates interstitial lung disease in SKG mice. Arthritis Res Ther.

[38] Chen Z, Wang X, Ye S (2019). Tofacitinib in amyopathic dermatomyositis-associated interstitial lung disease. N Engl J Med.

[39] Kurasawa K, Arai S, Namiki Y, Tanaka A, Takamura Y, Owada T (2018). Tofacitinib for refractory interstitial lung diseases in anti-melanoma differentiation-associated 5 gene antibody-positive dermatomyositis. Rheumatology.

[40] Kato M, Ikeda K, Kageyama T, Kasuya T, Kumagai T, Furuya H (2021). Successful treatment for refractory interstitial lung disease and pneumomediastinum with multidisciplinary therapy including tofacitinib in a patient with anti-MDA5 antibody-positive dermatomyositis. J Clin Rheumatol.

[41] Saldarriaga-Riuera LM, López-Villegas VJ (2019). Janus kinase inhibitors as a therapeutic option in rheumatoid arthritis and associated interstitial lung disease. Report of four cases. Rev Colomb Reumatol.

[42] Courvoisier DS, Chatzidionysiou K, Mongin D, Lauper K, Mariette X, Morel J (2021). The impact of seropositivity on the effectiveness of biologic anti-rheumatic agents: results from a collaboration of 16 registries. Rheumatology.

[43] Sokolove J, Schiff M, Fleischmann R, Weinblatt ME, Connolly SE, Johnsen A (2016). Impact of baseline anti-cyclic citrullinated peptide-2 antibody concentration on efficacy outcomes following treatment with subcutaneous abatacept or adalimumab: 2-year results from the AMPLE trial. Ann Rheum Dis.

[44] Chiu YH, Spierings J, de Jong PA, Hoesein FM, Grutters JC, van Laar JM (2021). Predictors for progressive fibrosis in patients with connective tissue disease associated interstitial lung diseases. Respir Med.

[45] Kawamura K, Ichikado K, Ichiyasu H, Anan K, Yasuda Y, Suga M (2019). Acute exacerbation of chronic fibrosing interstitial pneumonia in patients receiving antifibrotic agents: incidence and risk factors from real-world experience. BMC Pulm Med.

[46] Harigai M (2019). Growing evidence of the safety of JAK inhibitors in patients with rheumatoid arthritis. Rheumatology.

