# Supplementary material for: Janus kinase inhibitors vs. abatacept about safety and efficacy for patients with rheumatoid arthritis-associated interstitial lung disease: a retrospective nested case-control study
Source: BMC Rheumatol. 2024 Jan 26;8:4. doi: 10.1186/s41927-024-00374-x (PMC10811846; doi:10.1186/s41927-024-00374-x)
Supplement: Supplementary file 1 — Supplementary Material 1 [file 41927_2024_374_MOESM1_ESM.docx]

Supplementary Table 1. Scoring of interstitial pneumonia by computed tomography

| Score | | Feature |
| --- | --- | --- |
| GGO | 0 | No GGO |
|  | 1 | <5 % |
|  | 2 | 5 to <25 % |
|  | 3 | 25 to <50 % |
|  | 4 | 50 to <75 % |
|  | 5 | ≥75 % |
| Fibrosis | 0 | No fibrotic lesion |
|  | 1 | Interlobular septal thickening; no discrete honeycombing |
|  | 2 | 5 to <25 % |
|  | 3 | 25 to <50 % |
|  | 4 | 50 to <75 % |
|  | 5 | ≥75 % |

GGO: ground-glass opacities.

Supplementary Table 2. Total CT scores at the beginning of the treatment

A. CT scores of ABT group. B. CT scores of JAKi group. The full score is 15.

A

|  | GGO score | Fibrosis score |
| --- | --- | --- |
| Patient 1 | 3.5 | 1.5 |
| Patient 2 | 2 | 1 |
| Patient 3 | 6.5 | 0.5 |
| Patient 4 | 3.5 | 2 |
| Patient 5 | 2.5 | 4.5 |
| Patient 6 | 3.5 | 2.5 |
| Patient 7 | NA | NA |
| Patient 8 | 2.5 | 2.5 |
| Patient 9 | 5 | 3 |
| Patient 10 | 2 | 0.5 |
| Patient 11 | 2 | 5.5 |
| Patient 12 | 2 | 2.5 |
| Patient 13 | 5 | 0.5 |
| Patient 14 | 4 | 6 |
| Patient 15 | 3 | 1 |
| Patient 16 | 3.5 | 2 |
| Patient 17 | 3.5 | 3 |
| Patient 18 | 2 | 3 |
| Patient 19 | 5 | 3 |
| Patient 20 | NA | NA |
| Patient 21 | 3.5 | 6.5 |

B

|  | GGO score | Fibrosis score |
| --- | --- | --- |
| Patient 1 | 2.5 | 0.5 |
| Patient 2 | 6 | 5 |
| Patient 3 | 4 | 5.5 |
| Patient 4 | 7 | 7.5 |
| Patient 5 | 4.5 | 3.5 |
| Patient 6 | 4 | 2 |
| Patient 7 | 7 | 6 |
| Patient 8 | 4.5 | 4.5NA |
| Patient 9 | 3.5 | 4 |
| Patient 10 | NA | NA |
| Patient 11 | NA | NA |
| Patient 12 | 3.5 | 1.5 |
| Patient 13 | 5.5 | 5 |
| Patient 14 | 5 | 5 |
| Patient 15 | 4 | 5 |
| Patient 16 | 4 | 1.5 |
| Patient 17 | 5 | 3.5 |
| Patient 18 | NA | NA |
| Patient 19 | 4 | 0.5 |
| Patient 20 | NA | NA |
| Patient 21 | 4.5 | 1 |

ABT: abatacept; JAKi: Janus kinase inhibitor; GGO: ground-glass opacities; NA: Not available
